# Global and regional amyloid-β in explaining APOE-ε4 and sex associations with medial temporal tau: a neuropathology study

**DOI:** 10.1007/s00401-026-03095-2

**Published:** 2026-10-09

**Authors:** Gemma Salvadó, Colin Groot, Laura E. Wisse, Jacob W. Vogel, Thomas G. Beach, Geidy E. Serrano, Eric M. Reiman, Julie A. Schneider, David A. Bennett, Rik Ossenkoppele, Oskar Hansson

**Affiliations:** 1 Clinical Memory Research Unit, Department of Clinical Sciences Malmö, Lund University, Lund, Sweden; 2 Barcelonaβeta Brain Research Center (BBRC), Pasqual Maragall Foundation, Barcelona, Spain; 3 Hospital del Mar Research Institute, Barcelona, Spain; 4 Alzheimer Center Amsterdam, Neurology, Amsterdam UMC Location VUmc, Amsterdam, The Netherlands; 5 Amsterdam Neuroscience, Neurodegeneration, Amsterdam, The Netherlands; 6 Department of Clinical Sciences Lund, Lund University, Lund, Sweden; 7 Department of Clinical Sciences Malmö, SciLifeLab, Lund University, Lund, Sweden; 8 Banner Sun Health Research Institute, Sun City, AZ, USA; 9 Banner Alzheimer’s Institute, Arizona State University and University of Arizona, Phoenix, AZ USA; 10 Rush Alzheimer’s Disease Center, Rush University Medical Center, Chicago, IL USA; 11 Department of Neurological Sciences, Rush University Medical Center, Chicago, IL USA; 12 Memory Clinic, Skåne University Hospital, Malmö, Sweden; 13 Centro de Investigación Biomédica En Red - Fragilidad y Envejecimiento Saludable (CIBER-FES), Madrid, Spain

**Keywords:** Medial temporal lobe, APOE, Gender, Tangles, Plaques

## Abstract

The medial temporal lobe (MTL) is a key region in Alzheimer’s disease (AD) pathogenesis, affected early by tau pathology. While female sex and *APOE-ε4* are two known risk factors that affect tau levels, whether these factors drive tau in the MTL via amyloid-dependent or amyloid-independent mechanisms remains unclear. We investigated these relationships in two large neuropathological cohorts comprising 2550 participants from the Religious Orders Study/Memory and Aging Project (ROS/MAP; *n*=1587, main cohort) and Arizona Study of Aging and Neurodegenerative Disorders (AZSAND; n=963, replication cohort) covering the whole AD continuum. Neuropathological assessments of global neocortical and regional MTL amyloid-β burden were used to determine the extent to which amyloid-β pathology mediated associations of *APOE-ε4* carriership and sex with MTL tau burden. In both cohorts, *APOE-ε4* carriership and female sex were associated with significantly greater MTL tau pathology (all *p*<0.001). Adjustment for global amyloid-β attenuated but did not eliminate these associations (*p*≤0.024). However, after jointly accounting for global and MTL amyloid-β burden, the association between *APOE-ε4* and MTL tau was no longer significant in either cohort (*p*≥0.19), whereas the association between female sex and MTL tau remained robust (*p*≤0.004). Models incorporating both global and regional MTL amyloid-β explained substantially more variance in MTL tau burden than models including only global amyloid-β, and results were replicated when further adjusting for diagnostic group or neocortical tau burden. These findings suggest that *APOE-ε4* and female sex influence MTL tau accumulation through partially distinct pathogenic pathways, supporting an additional contribution of regional amyloid-β to the APOE-ε4-associated MTL tau burden while implicating additional amyloid-independent mechanisms in female vulnerability to tau accumulation.

**Supplementary Information:**

The online version contains supplementary material available at https://doi.org/10.1007/s00401-026-03095-2.

## Introduction

The medial temporal lobe (MTL) plays a key role in Alzheimer’s disease (AD). The MTL is one of the first brain regions to accumulate tau pathology [11], and this process is closely related to episodic memory impairment typical in AD patients [27]. While the appearance of tau pathology in neocortical brain regions is strongly associated with the presence amyloid-β pathology [48, 56], the relationship between MTL tau and amyloid-β pathology is less well understood [43]. Aside from AD, age-related mechanisms have also been implicated in MTL tau accumulation, even in the absence of amyloid-β pathology [12, 51, 54, 70]. However, how two other established AD risk factors [58], *APOE-ε4* carriership and female sex, relate to MTL tau and amyloid-β (and their interplay) remains unclear.

*APOE-ε4* carriership is a well-established genetic risk factor for amyloid-β pathology [39, 41], whereas sex differences in amyloid-β are less consistent [1, 14]. On the other hand, both APOE-ε4 carriership and female sex have been associated with higher tau levels across modalities, including neuropathology [4, 55], tau-PET [16, 22, 54, 57, 59, 68], and CSF biomarkers [15, 22, 37]. Animal and in vitro studies suggest an amyloid-β-independent effect of *APOE-ε4* on tau pathology [43, 45, 63], with neuroimaging studies suggesting that this effect might be restricted to the MTL [59, 68]. Importantly, prior imaging studies have typically assessed amyloid-β using global neocortical composites, potentially overlooking local amyloid-β and tau interplay in the MTL.

Unraveling the direct and amyloid-β-mediated effects of *APOE-ε4* carriership and sex on tau has important implications for understanding disease progression. These findings may also inform clinical trials, improving enrichment strategies for therapies that target amyloid-β, tau or APOE biology [25]. Although PET studies have provided important insights into *APOE-ε4*- and sex-related effects on amyloid-β and tau [22, 54, 59, 68], assessing pathology within the MTL remains challenging because of limited spatial resolution, off-target binding, and reduced sensitivity in this region [34, 53]. In contrast, neuropathological assessment provides higher sensitivity and specificity to quantify tau in this specific region. Thus, the main goal of this study was to investigate the direct and amyloid-β-mediated associations of *APOE-ε4* carriership and sex with neuropathologically characterized tau in the MTL.

## Methods

### Participants

Participants included in this study came from two independent neuropathological datasets: the ROS/MAP (main cohort) and the Arizona Study of Aging and Neurodegenerative Disorders (AZSAND, replication cohort). ROS/MAP participants come from two longitudinal clinicopathological community-based cohort studies of aging and dementia, the Religious Orders Study (ROS) and the Rush Memory and Aging Project (MAP), which have been described previously [8]. In brief, ROS started recruiting older Catholic nuns, priests, and brothers from sites across the USA in 1994. MAP has been enrolling participants across the metropolitan area of Chicago since 1997. Both studies were approved by an Institutional Review Board at Rush University Medical Center. All participants signed informed and repository consents and an Anatomic Gift Act. At baseline, all participants were without known dementia. As both studies have a large common core of data identical at the item level collected by the same team with the same trainer, we treat them as a single, combined cohort (ROS/MAP) from here onward. The other neuropathological dataset included participants from AZSAND Brain and Body Donation Program (enrolled between 2007 and 2019), which has been also described previously [6]. The AZSAND cohort recruited cognitively unimpaired participants but also put specific efforts into recruiting participants diagnosed with AD, Parkinson’s disease, and cancer. Due to the expected difference in tau distribution in early-onset dementia (i.e., higher chance of MTL-sparing tau patterns [21, 52, 69]), we restricted our analyses to participants with an AD diagnosis at age equal or above 65 years [31].

### Neuropathological measures

#### Amyloid-β and tau

Measures of amyloid-β load and tau tangle density in ROS/MAP were obtained as previously described [9, 10, 60]. In brief, amyloid-β deposits and tau tangle pathologies were assessed using MO0872 and AT8, respectively. Aβ load was quantified as the percentage of tissue area occupied by Aβ immunoreactivity, whereas tau pathology was quantified as neurofibrillary tangle density. These measures were obtained across eight cortical regions: including the entorhinal cortex, hippocampus (CA1/subiculum), superior frontal cortex (Brodmann area [BA] 6/8), mid-frontal cortex (BA 46/9), inferior temporal cortex (BA 20), angular gyrus (BA 39/40), cingulate cortex (BA 32/33), and calcarine cortex (BA 17). For this study, we created a MTL composite by averaging measures of the entorhinal cortex and hippocampus for both amyloid-β and tau pathology. To obtain neocortical amyloid-β and tau composites, we averaged measures of the remaining six regions. In addition to these immunohistochemical measures, diffuse and neuritic plaques were assessed separately by microscopic examination of silver-stained sections. Diffuse plaques were defined as deposits showing a diffuse blush or filamentous appearance without distended or abnormal neurites and without a compact amyloid core, whereas neuritic plaques were characterized by distended or abnormal neurites, with or without a central amyloid core. Unless otherwise specified, references to amyloid-β plaque pathology in the analyses of the present study refer to neuritic plaque burden.

Methods for the semi-quantification of amyloid-β and tau in the AZSAND dataset have been described previously [7]. In brief, amyloid-β plaque and neurofibrillary tangle density were measured using a semi-quantitative scale following the Consortium to Establish a Registry for Alzheimer’s Disease (CERAD) [50] templates (i.e., none, sparse, moderate and frequent) following staining with thioflavin-S, Campbell–Switzer and Gallyas methods. Total amyloid-β burden including cored, neuritic, and diffuse plaques was derived from Campbell–Switzer staining, whereas thioflavin-S staining was used to estimate neuritic plaque density. Neurofibrillary pathology was evaluated across all three staining methods, as each highlights neurofibrillary changes. These categorizations were then transformed into a scale from 0 to 3 in each of the regions investigated. These regions included the frontal, the temporal, the parietal and the entorhinal cortices, and the hippocampus. Combining entorhinal and hippocampus scores, we created an MTL measure for each pathology that ranged from 0 to 6. A neocortical global measure was created for each pathology by summing scores from the frontal, temporal, and parietal cortices, yielding a total score ranging from 0 to 9.

#### Co-pathologies

The possibility of measuring various co-pathologies on top of amyloid-β and tau is one of the main strengths of neuropathological data relative to neuroimaging data. We therefore adjusted our analyses for the presence of TDP-43, Lewy-body pathology and vascular lesions to minimize confounding effects (see statistical analyses). More specifically, in the ROS/MAP dataset, TDP-43 was assessed in five brain regions using staining with monoclonal antibodies to phosphorylated TDP-43 (pS409/410; 1:100) as previously described [36]. A dichotomized measure was created with TDP-43 being assessed as positive if there was pathology beyond the amygdala. The presence of Lewy-body pathology (LBP) was assessed using a monoclonal antibody to detect α-synuclein (Zymed LB 509; 1:50). LBP was considered positive if it was either limbic type or neocortical type. The presence of gross and microscopic infarcts was also assessed as previously described [61]. Atherosclerosis, arteriosclerosis, and cerebral amyloid angiopathy (CAA) were all rated on a 4-point scale (0–3) from none to severe and then dichotomized as positive (≥ 1) vs negative (0) for all features independently [3].

In the AZSAND dataset, CAA was graded on a 0–3 scale based analogously on CERAD templates [50] and assessed as positive if the score was ≥1. LBP was measured with immunohistochemical staining for p129 alpha-synuclein and thioflavin-S in 10 brain regions based on a Unified Staging System [5]. TDP-43 measurements were only available in a subset of participants and were measured by immunohistochemistry of the mesial temporal lobe using an antibody against phosphorylated TDP-43 peptide, as previously detailed [2].

### Statistical analyses

We performed our main analyses in ROS/MAP and we used the AZSAND dataset as a replication cohort. First, we investigated the effects of *APOE-ε4* carriership and sex on MTL tau using a linear regression model adjusting for age, and presence of co-pathologies in a single model per cohort (Model 1). All available co-pathologies were initially tested, and only those associated with MTL tau (p<0.05) were retained in subsequent models. Based on this criterion, for both ROS/MAP and the AZSAND datasets, CAA and LBP were used as covariates, and in ROS/MAP, we included atherosclerosis in the model. Although our main interest was in MTL tau, we also investigated main effects of sex and *APOE-ε4* carriership on amyloid-β pathology in the MTL and in the neocortex.

Next, we aimed to investigate whether sex and *APOE-ε4* effects on MTL tau were mediated by *global* (i.e., neocortical) and/or *regional* (i.e., MTL) amyloid-β load. To assess mediation by amyloid-β, we added global amyloid-β (Model 2), MTL amyloid-β (Model 3), or both measures simultaneously (Model 4) as covariates. In ROS/MAP, a fifth model additionally included neocortical diffuse amyloid-β (Model 5) which was not available in the AZSAND cohort. To account for possible collinearity arising from the inclusion of multiple amyloid-β measures in the models, we calculated the variance inflation factor (VIF) with the *vif* function in R using the *car* package. As a rule of thumb, a VIF>5 may be cause for concern, and a VIF>10 indicates serious problems of collinearity [49].

In a second step, we performed path analyses to investigate possible mediation effects of each amyloid-β measure in each cohort. The best models were selected based on having the lowest Akaike information criterion (AIC), always favoring the simpler models. Statistical differences between models were tested with an F-test (nested models) or Vuong’s test (non-nested models, *nonnest2* package). Next, we constructed structural equation models (SEM) to further explore the interconnections between all variables included in the optimal model, separately for each cohort, using the *lavaan* package. A single model per cohort was done with age, sex, *APOE-ε4* carriership and presence of co-pathologies as covariates [65]. Then, we included the amyloid-β measures as predictor and outcome variables following a sequential order going from diffuse neocortical plaques (only in ROS/MAP), to neocortical neuritic plaques, to MTL neuritic plaques to MTL neurofibrillary tau tangles (see Supplementary Table 2 for a detailed outline of the final model). This ordering was specified a priori based on neuropathological staging, with diffuse plaques preceding neuritic plaques [66] and Aβ deposition in the neocortex preceding involvement of the entorhinal cortex and hippocampus [67]. Percentage mediation (PM) effect for these paths was expressed by the percentage of the total effect (i.e., calculated without any amyloid-β measures in the model). Goodness-of-fit was assessed using the comparative fit index (CFI), Tucker–Lewis index (TLI), and the root mean square error of approximation (RMSEA). Good model fit was defined as CFI>0.95, TLI>0.95, and RMSEA<0.05 [38].

All analyses were performed using R (v.4.1.0), and the statistical threshold for significant effects was set at p<0.05 (two-sided).

## Results

The ROS/MAP dataset included 369 (23.3%) *APOE-ε4* carriers and 1,076 (67.8%) females, and the AZSAND dataset included 369 (38.3%) *APOE-ε4* carriers and 413 (42.9%) females. Demographic and clinical characteristics are summarized in Table 1. Notably, both ROS/MAP and AZSAND datasets were mainly constituted of cognitively impaired individuals.

**Table 1:** Demographic characteristics by cohort

	ROS/MAP dataset					AZSAND dataset				
	Overall (*N*=1,587)	Male (*N*=511)	Female (*N*=1,076)	*APOE-ε4* Non-carriers (*N*=1218)	*APOE-ε4* Carriers (*N*=369)	Overall (*N*=963)	Male (*N*=550)	Female (*N*=413)	*APOE-ε4* Non-carriers (*N*=594)	*APOE-ε4* Carriers (*N*=369)
Age at death, mean (SD)	89.7 (6.60)	88.3 (6.50)	90.3 (6.55)	90.1 (6.60)	88.4 (6.43)	83.3 (7.36)	82.3 (7.65)	84.7 (6.73)	83.5 (8.08)	83.1 (6.03)
Female, *n* (%)	1076 (67.8%)	–	1076 (100%)	829 (68.1%)	247 (66.9%)	413 (42.9%)	–	413 (100%)	262 (44.1%)	151 (40.9%)
*APOE-ε4* carriership, *n* (%)	369 (23.3%)	122 (23.9%)	247 (23.0%)	–	369 (100%)	369 (38.3%)	218 (39.6%)	151 (36.6%)	–	369 (100%)
Diagnosis, *n* (%)										
CU	521 (32.8%)	176 (34.4%)	345 (32.1%)	441 (36.2%)	80 (21.7%)	196 (20.4%)	102 (18.5%)	94 (22.8%)	158 (26.6%)	38 (10.3%)
MCI	365 (23.0%)	129 (25.2%)	236 (21.9%)	301 (24.7%)	64 (17.3%)	53 (5.5%)	31 (5.6%)	22 (5.3%)	41 (6.9%)	12 (3.3%)
AD	676 (42.6%)	197 (38.6%)	479 (44.5%)	458 (37.6%)	218 (59.1%)	410 (42.6%)	214 (38.9%)	196 (47.5%)	169 (28.5%)	241 (65.3%)
Other	25 (1.6%)	9 (1.8%)	16 (1.5%)	18 (1.5%)	7 (1.9%)	304 (31.5%)	203 (36.9%)	101 (24.5%)	226 (38.0%)	78 (21.1%)
Neuropathologic measures										
Neocortical diffuse amyloid-β plaques, mean (SD)^†^	13.2 (14.0)	12.6 (14.2)	13.5 (13.9)	11.4 (13.2)	19.3 (14.8)	–	–	–	–	–
Neocortical amyloid-β plaques, mean (SD)	11.0 (11.1)	9.11 (9.83)	11.9 (11.5)	9.31 (9.84)	16.6 (13.0)	5.93 (3.76)	5.62 (3.88)	6.35 (3.55)	4.73 (3.95)	7.88 (2.38)
MTL neuritic amyloid-β plaques, mean (SD)	7.54 (8.66)	5.64 (6.99)	8.44 (9.21)	6.19 (7.68)	12.0 (10.1)	2.98 (2.23)	2.86 (2.29)	3.14 (2.15)	2.23 (2.19)	4.18 (1.71)
MTL neurofibrillary tangles, mean (SD)	24.1 (21.6)	19.5 (19.3)	26.3 (22.3)	22.0 (20.2)	31.0 (24.6)	4.65 (1.73)	4.42 (1.83)	4.96 (1.54)	4.29 (1.82)	5.22 (1.39)
CAA, *n* (%)	555 (35.0%)	174 (34.1%)	381 (35.4%)	350 (28.7%)	205 (55.6%)	569 (59.1%)	314 (57.1%)	255 (61.7%)	265 (44.6%)	304 (82.4%)
LBP, *n* (%)	340 (21.4%)	120 (23.5%)	220 (20.4%)	246 (20.2%)	94 (25.5%)	233 (24.2%)	126 (22.9%)	107 (25.9%)	114 (19.2%)	119 (32.2%)
Arteriosclerosis, *n* (%)	503 (31.7%)	137 (26.8%)	366 (34.0%)	390 (32.0%)	113 (30.6%)	–	–	–	–	–
TDP-43, *n* (%)*	511 (33.8%)	137 (29.0%)	374 (36.0%)	354 (29.1%)	157 (45.5%)	126 (21.4%)	70 (21.7%)	56 (21.1%)	57 (15.4%)	69 (31.7%)

^†^Ten cases missing for ROS/MAP

^*^76 cases missing for ROS/MAP and 375 cases missing in AZSAND

Abbreviations: AD, Alzheimer’s dementia; AZSAND, Arizona Study of Aging and Neurodegenerative Disorders; CAA, cerebral amyloid angiopathy; CU, cognitively unimpaired; LBP, Lewy-body pathology; MCI, mild cognitive impairment; MTL, medial temporal lobe; ROS/MAP, Religious Orders Study and Rush Memory and Aging Project

### *APOE-ε4* carriership and female sex are associated with increased levels of MTL tau

**Fig. 1:**
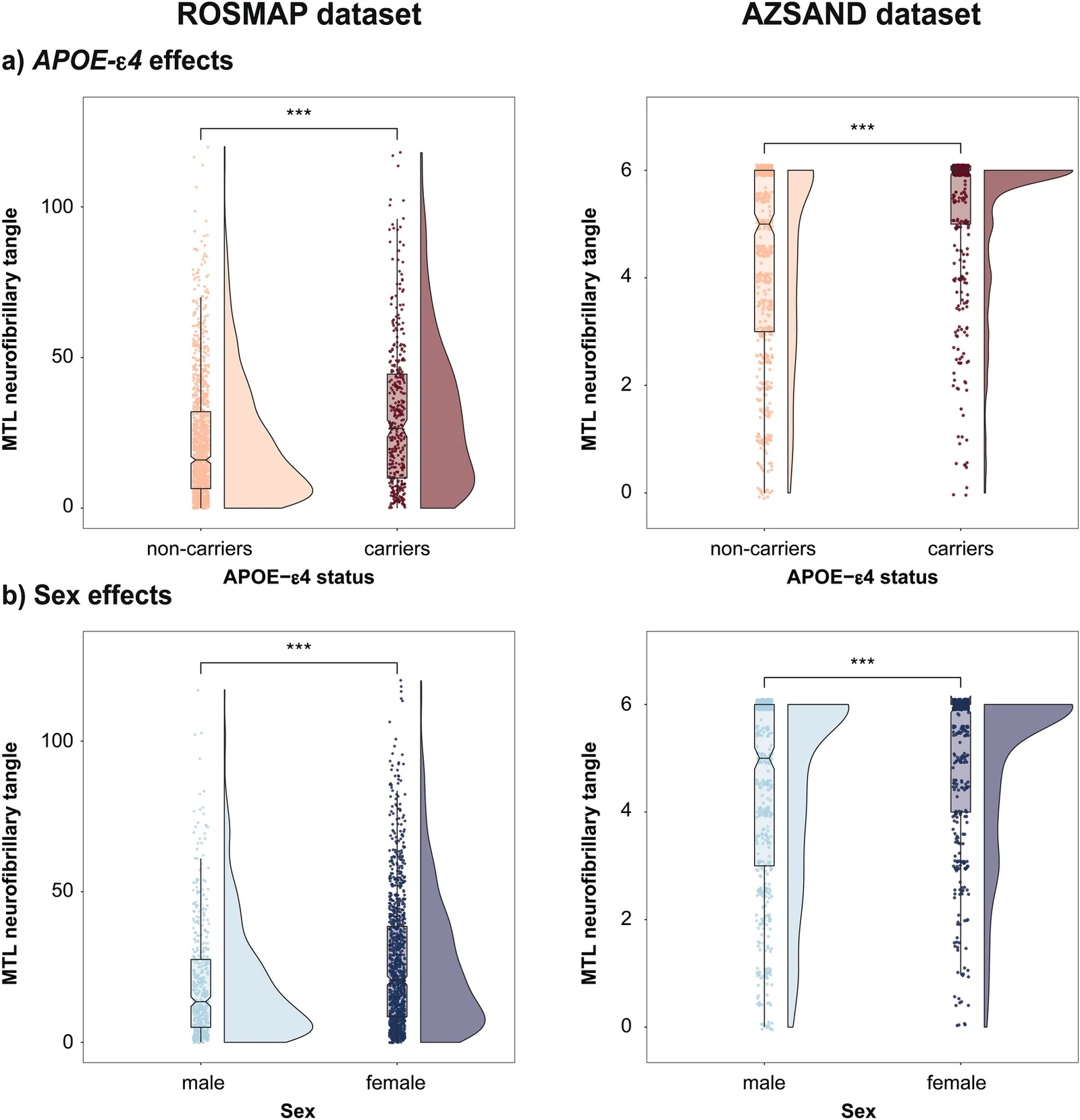
Total effects of *APOE-ε4* carriership and female sex on MTL tau. Differences on raw MTL neurofibrillary tau tangle measurements by *APOE-ε4* carriership (**A**) and female sex (**B**) in ROS/MAP (first column) and AZSAND (second column) datasets. Statistical differences are derived from linear regression models including both predictors in a single model adjusting for age and presence of other co-pathologies (ROS/MAP: CAA, LBP and atherosclerosis; AZSAND: CAA and LBP). Differences in scale are due to fully quantitative (ROS/MAP) and semi-quantitative (AZSAND) methods of pathology measurement. ***p<0.001. AZSAND, Arizona Study of Aging and Neurodegenerative Disorders; CAA, cerebral amyloid angiopathy; LBP, Lewy-body pathology; MTL, medial temporal lobe; ROS/MAP, Religious Orders Study and Rush Memory and Aging Project

**Fig. 2:**
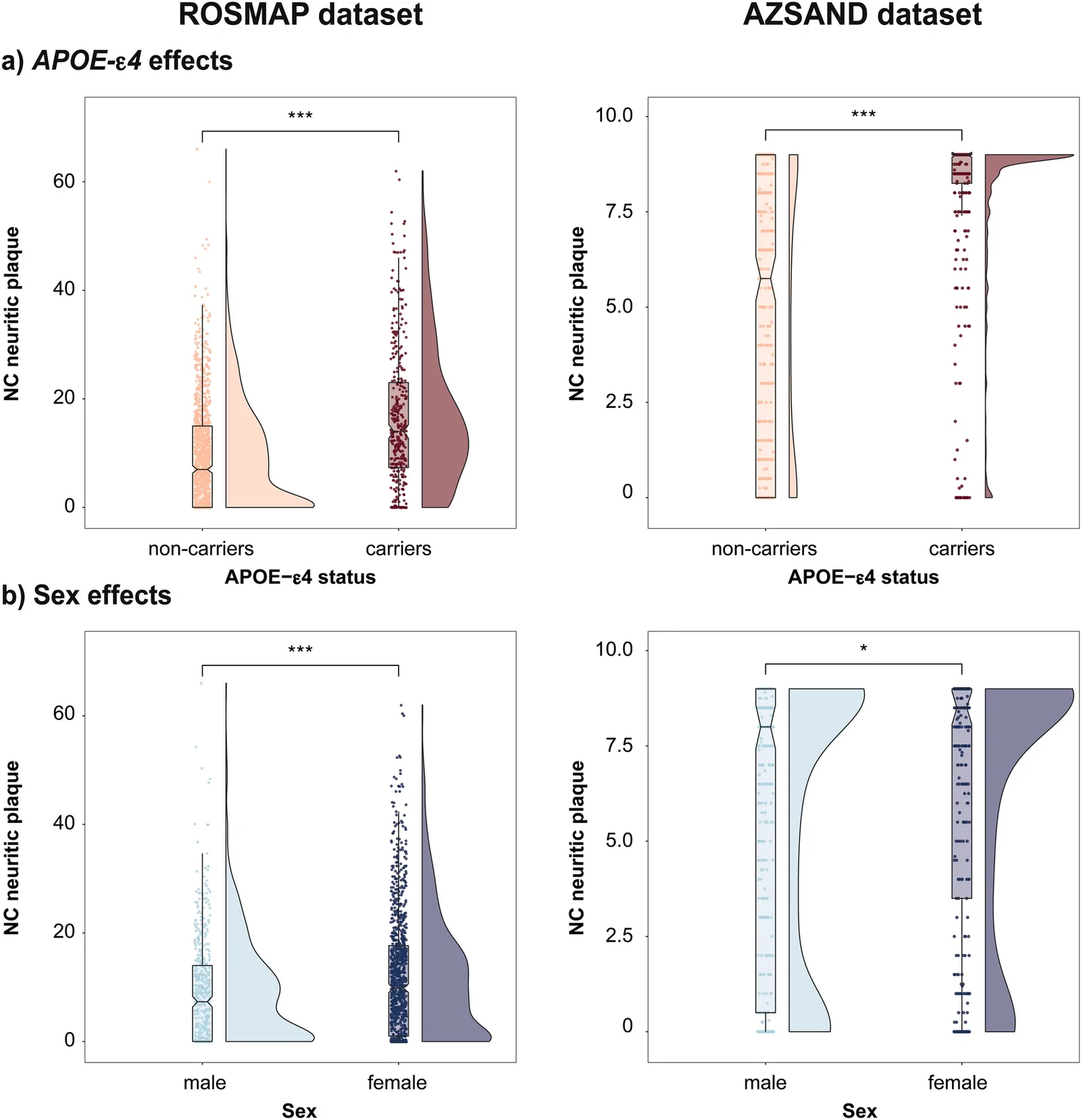
Sex and *APOE-ε4* carriership total effects on neocortical amyloid-β. Sex (**A**) and *APOE-ε4* carriership (**B**) effects on neocortical amyloid-β in ROS/MAP (left column) and AZSAND (right column) datasets. Both effects are calculated from the same model. *P*-values were calculated adjusting for age and presence of other co-pathologies (ROS/MAP: CAA, LB and atherosclerosis and, Arizona: CAA and LB). *p<0.05; ***p<0.001**.** AZSAND, Arizona Study of Aging and Neurodegenerative Disorders; CAA, cerebral amyloid angiopathy; LB, Lewy-body pathology; NC, neocortical; ROS/MAP, Religious Orders Study and Rush Memory and Aging Project

**Fig. 3:**
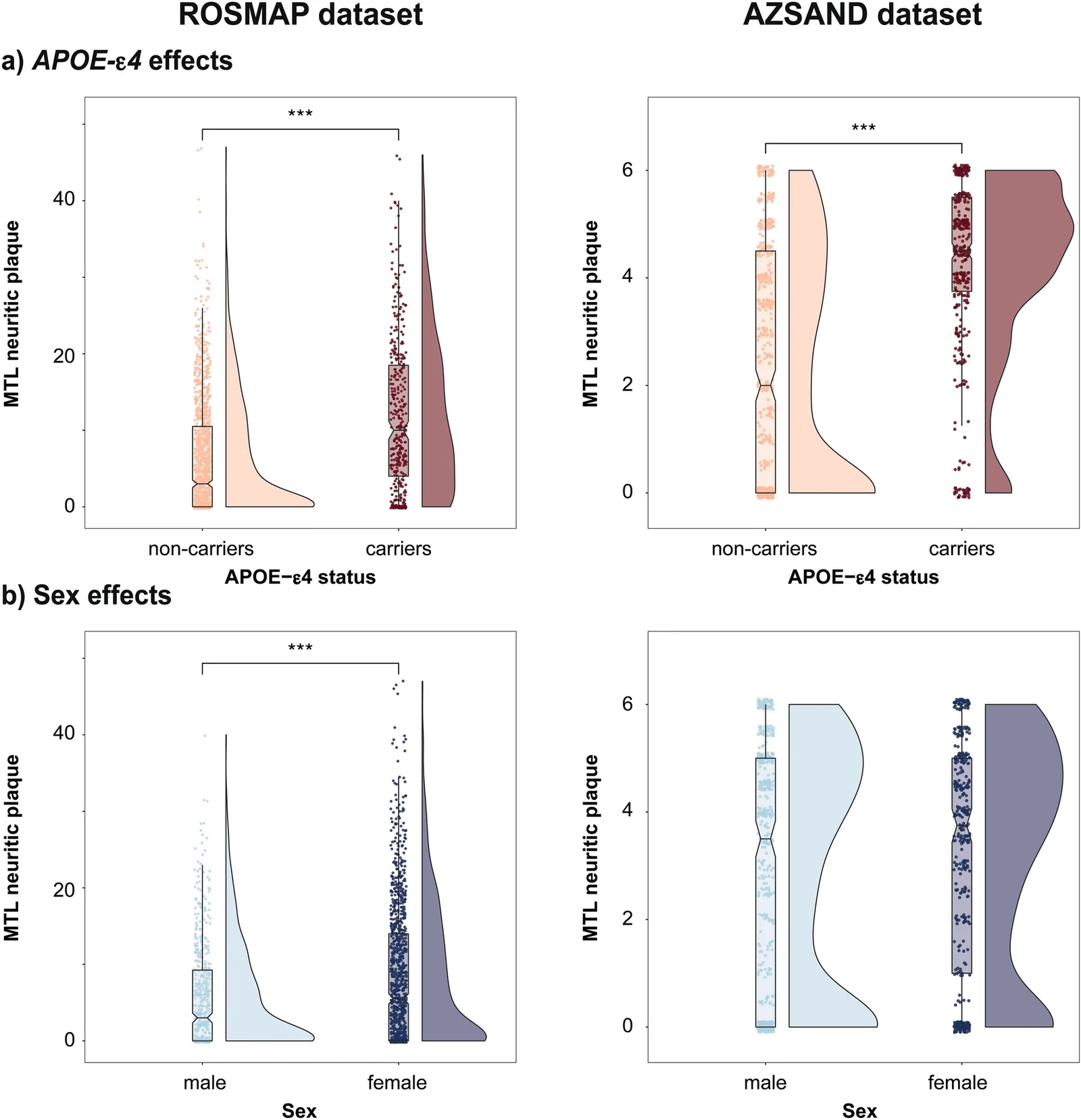
Sex and *APOE-ε4* carriership total effects on MTL amyloid-β. Differences on raw MTL amyloid-β measurements by *APOE-ε4* carriership (**A**) and female sex (**B**) in ROS/MAP (first column) and AZSAND (second column) datasets. P-values were calculated adjusting for age and presence of other co-pathologies (ROS/MAP: CAA, LB and atherosclerosis; Arizona: CAA and LB). ***p<0.001. AZSAND, Arizona Study of Aging and Neurodegenerative Disorders; CAA, cerebral amyloid angiopathy; LB, Lewy-body pathology; MTL, medial temporal lobe; ROS/MAP, Religious Orders Study and Rush Memory and Aging Project

We first investigated the effects of *APOE-ε4* carriership and female sex on global and MTL tau and MTL amyloid-β (without adjusting for amyloid-β measures). Confirming previous literature, both *APOE-ε4* carriership (ROS/MAP: β[95% CI]=0.35[0.24, 0.47]; AZSAND: β[95% CI]=0.32[0.20, 0.43], both *p*<0.001) and sex (ROSMAP: β[95% CI]=0.27[0.17, 0.37]; AZSAND: β[95% CI]=0.20[0.09, 0.30], both p<0.001, Table 2) had a significant effect on MTL tau, with *APOE-ε4* carriers and females showing higher tau burden in the MTL across cohorts (Fig. 1). Both *APOE-ε4* carriership and female sex also had a significant positive effect on neocortical amyloid-β in both cohorts (Supplementary Table 1 and Fig. 2). For amyloid-β load in the MTL, *APOE-ε4* carriership showed a positive association in both cohorts (ROS/MAP: β[95% CI]=0.59[0.48, 0.70]; AZSAND: β[95% CI]=0.51[0.40, 0.62], both *p*<0.001), but the female sex effect on MTL amyloid-β only reached statistical significance in the ROS/MAP dataset (ROS/MAP: β[95% CI]= 0.32[0.22, 0.42], *p*<0.001; AZSAND: β[95% CI]=0.07[− 0.03, 0.17], *p*=0.178, Supplementary Table 1 and Fig. 3). Notably, similar results were observed when adjusting for global amyloid-β. The *APOE-ε4* carriership effects on MTL amyloid-β remained significant in both cohorts (ROS/MAP: β[95% CI]=0.20[0.12, 0.28]; AZSAND: β[95% CI]= 0.10[0.04, 0.16], both *p*≤0.002) but female sex effects (ROS/MAP: β[95% CI]= 0.15[0.07, 0.22], *p*<0.001; AZSAND: β[95% CI]= − 0.03[− 0.09, 0.02], *p*=0.214) were only significant in the in the ROS/MAP cohort (Supplementary Table 1).

**Table 2:** *APOE-ε4* carriership and sex effects on MTL tau

	ROS/MAP dataset						AZSAND dataset					
	Female sex		*APOE-ε4* carriership		*R*^2^	AIC	Female sex		*APOE-ε4* carriership		*R*^2^	AIC
	β [95% CI]	*p*	*β* [95% CI]	*p*			*β* [95% CI]	*p*	*β* [95% CI]	*p*		
Model 1	0.27 [0.17, 0.37]	<0.001	0.35 [0.24, 0.47]	<0.001	0.12	4282	0.20 [0.09, 0.30]	<0.001	0.32 [0.20, 0.43]	<0.001	0.30	2402
Model 2	0.17 [0.08, 0.27]	<0.001	0.14 [0.03, 0.25]	0.011	0.25	4042	0.15 [0.05, 0.25]	0.004	0.13 [0.02, 0.25]	0.024	0.38	2279
Model 3	0.14 [0.04, 0.23]	0.006	0.11 [0.00, 0.22]	0.048	0.27	3986	0.16 [0.07, 0.26]	0.001	0.08 [− 0.04, 0.19]	0.185	0.43	**2196**
Model 4	0.13 [0.04, 0.22]	0.005	0.08 [− 0.02,0.19]	0.121	0.28	3961	0.17 [0.07, 0.27]	0.001	0.08 [− 0.03, 0.19]	0.159	0.43	2196
Model 5	0.14 [0.04, 0.23]	0.004	0.07 [− 0.04, 0.18]	0.209	0.29	**3957**	–	–	–	–	–	–

Model 1 shows the total female sex and *APOE-ε4* carriership effect (only adjusted for covariates) on MTL tau, while Models 2–5 represent direct effects (independent of amyloid-β burden). Model 2 and Model 3 are adjusted for global (neocortical) or regional (MTL) amyloid-β burden, respectively, and Model 4 includes the two amyloid-β measures. Finally, in the ROS/MAP dataset, we included a fifth model (Model 5) additionally adjusting for diffuse amyloid-β burden. In the ROS/MAP cohort, all amyloid-β measures refer to neuritic plaques unless otherwise stated. All models include age and presence of co-pathologies as covariates (ROS/MAP: CAA, LBP and atherosclerosis; AZSAND: CAA and LBP). Each row is a unique model including both sex and *APOE-ε4* carriership as predictors in the same model, per cohort. Males and *APOE-ε4* non-carriers are the reference group. R^2^ is adjusted for the number of terms in the model. Best models (shown in bold) per cohort were selected based on AIC and statistical comparison among models using F-tests

AIC, Akaike information criterion; AZSAND, Arizona Study of Aging and Neurodegenerative Disorders; CAA, cerebral amyloid angiopathy; CI, confidence interval; LBP, Lewy-body pathology; MTL, medial temporal lobe; NFT, neurofibrillary tangles; ROS/MAP, Religious Orders Study and Rush Memory and Aging Project

### Direct and amyloid-β-mediated effects of *APOE-ε4* carriership and female sex on MTL tau

To investigate the direct effects of *APOE-ε4* carriership and female sex on MTL tau, we first performed linear regression models that included global and/or regional amyloid-β measures as covariates. In both cohorts, after adjusting for global amyloid-β, the effects of *APOE-ε4* carriership (ROS/MAP: β[95% CI]=0.14 [0.03, 0.25], *p*=0.011; AZSAND: β[95% CI]=0.13 [0.02, 0.25], *p*=0.024) and female sex (ROS/MAP: β[95% CI]= 0.17 [0.08, 0.27], *p*<0.001; AZSAND: β[95% CI]= 0.15 [0.05, 0.25], *p*=0.004) on MTL tau remained significant, although were lower than without adjustment.

**Fig. 4:**
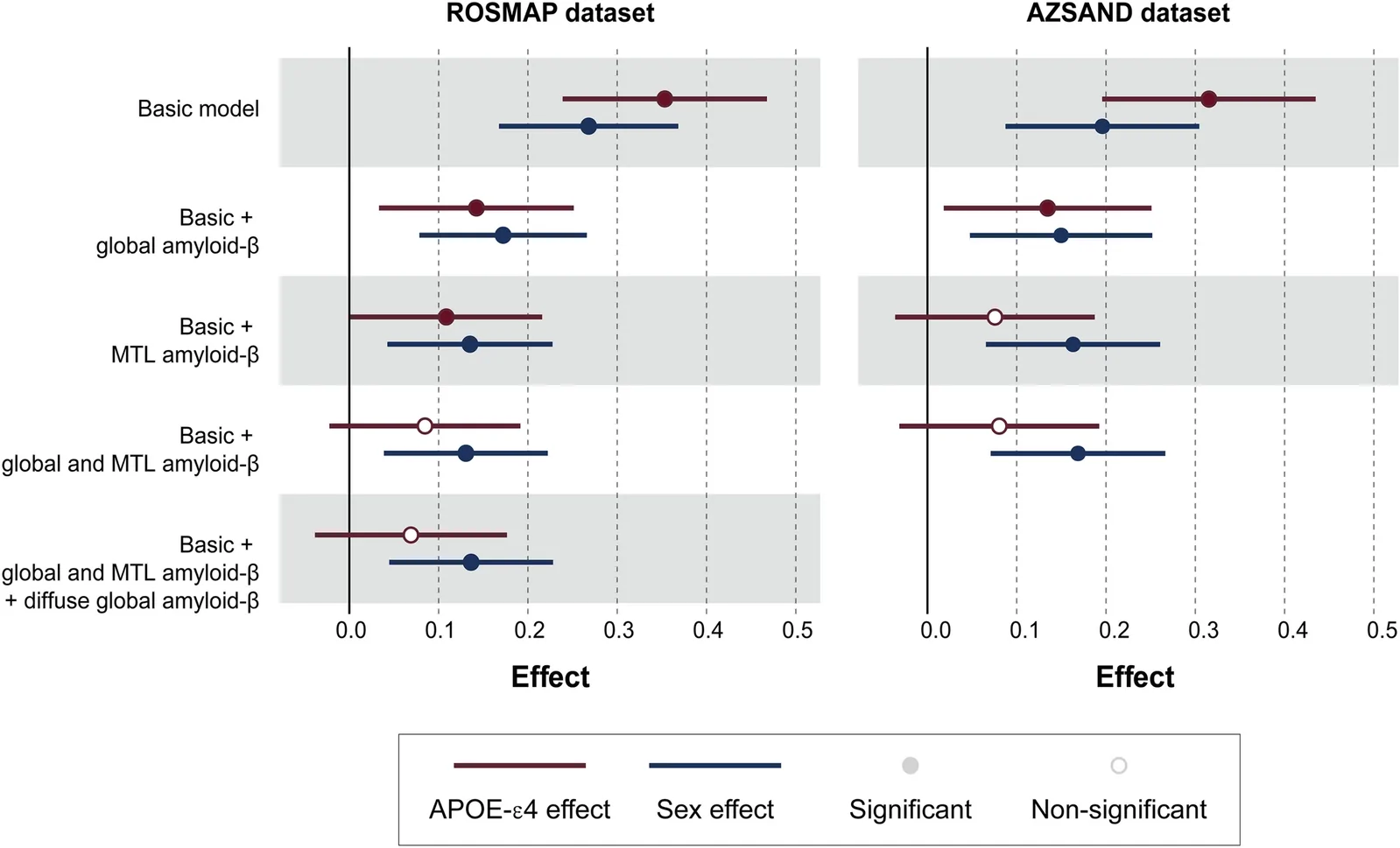
Direct effects of *APOE-ε4* carriership and female on MTL tau. *APOE-ε4* carriership (dark red) and female sex (dark blue) effects on MTL neurofibrillary tau tangles in ROS/MAP (**A**) and AZSAND (**B**) datasets, in different statistical models. Both effects are calculated from a single model in each case. Model 1 shows the total *APOE-ε4* carriership and female sex effects on MTL tau, while Models 2–5 represent direct effects (independent of amyloid-β burden). Model 2 and Model 3 are adjusted for global (neocortical) and regional (MTL) amyloid-β burden, respectively; Model 4 includes the two amyloid-β measures. Finally, in the ROS/MAP dataset, we included a fifth model (Model 5) additionally adjusting for neocortical diffuse amyloid-β burden. All models included age and presence of other co-pathologies (ROS/MAP: CAA, LBP and atherosclerosis; AZSAND: CAA and LBP) as covariates. Filled (empty) dots represent significant (non-significant) effects, and bar length corresponds to the 95% CI. AZSAND, Arizona Study of Aging and Neurodegenerative Disorders; CAA, cerebral amyloid angiopathy; CI, confidence intervals; LBP, Lewy-body pathology; MTL, medial temporal lobe; ROS/MAP, Religious Orders Study and Rush Memory and Aging Project

Based on the AIC, the best model in the ROS/MAP dataset included neocortical diffuse and neuritic amyloid-β plaques, and MTL neuritic plaques (Model 5: *R*^2^=0.29, AIC=3,940, Table 2 and Fig. 4). In this model, we observed a significant female sex effect on MTL tau (β[95% CI]=0.14 [0.04, 0.23], *p*=0.004) but the *APOE-ε4* carriership effect was no longer significant (β[95% CI]=0.07 [− 0.04, 0.18], *p*=0.209). In the AZSAND dataset, the model only including MTL amyloid-β was selected as optimal based on the AIC criterion (Model 3: *R*^2^=0.43, AIC=2196), although the results were very similar when additionally adding the neocortical amyloid-β metric (Model 4: *R*^2^=0.46, AIC=2,196, p_models_=0.144). Similar to the ROS/MAP analyses, the optimal model included a significant effect of female sex (β[95% CI]=0.16 [0.07, 0.26], *p*=0.001), but the effect of *APOE-ε4* carriership was not significant (β[95% CI]=0.08 [− 0.04, 0.19], *p*=0.185). The correlations between both amyloid-β measures was high in both cohorts (ROS/MAP: Spearman’s ρ=0.82; AZSAND: *ρ*=0.87), but the VIF was all below 5, supporting the model’s validity as there was no indication of collinearity [40, 49].

**Fig. 5:**
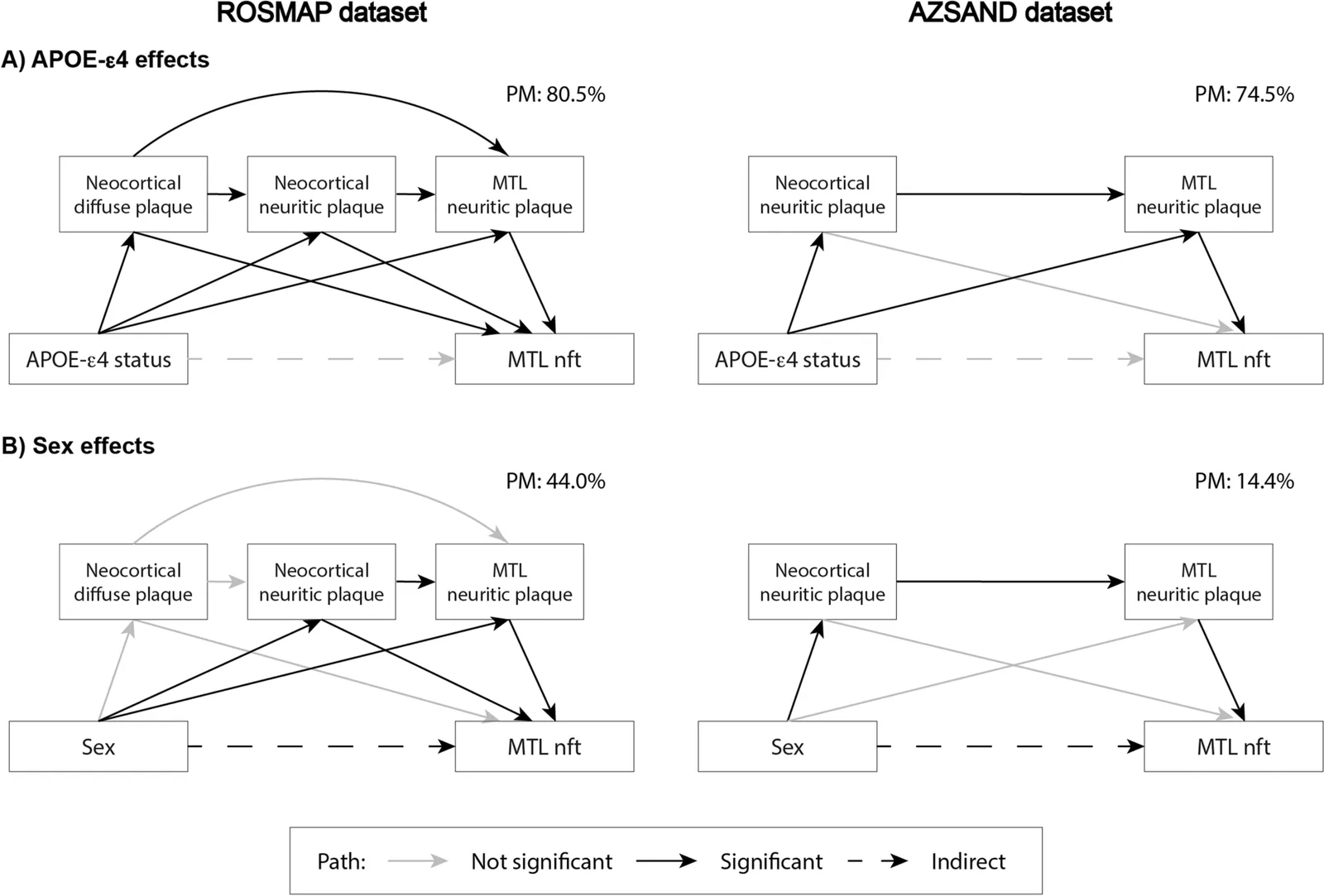
Schematic direct and amyloid-β-mediated effects of *APOE-ε4* carriership and female on MTL tau. Schematic depiction of the direct and amyloid-β-mediated *APOE-ε4* carriership (**A**) and female sex (**B**) effects on MTL tau in ROS/MAP (first column) and AZSAND (second column) datasets. Both effects are calculated from a single path analysis, adjusting for age and presence of other co-pathologies (ROS/MAP: CAA, LBP and atherosclerosis; AZSAND: CAA and LBP), but are depicted separately for visual purposes. Black (light grey) lines represent significant (non-significant) paths and dashed lines represent the direct effect after adjusting for all the amyloid-β measures. It can be observed that the direct female sex effect, but not the *APOE-ε4* carriership direct effect, on MTL tau was significant in both cohorts. Percentage mediation (PM) effect was calculated as the percentage of the total effect that was mediated by amyloid-β measures. AZSAND, Arizona Study of Aging and Neurodegenerative Disorders; CAA, cerebral amyloid angiopathy; LBP, Lewy-body pathology; MTL, medial temporal lobe; PM, percentage mediation; ROS/MAP, Religious Orders Study and Rush Memory and Aging Project

In a next step, we used a path analysis approach to investigate the direct and indirect effects of *APOE-ε4* carriership and female sex on MTL tau. To this end, we included amyloid-β measures as mediators in a sequential order being: diffuse neocortical (only in ROS/MAP), neuritic neocortical, and neuritic MTL amyloid-β plaques (Fig. 5), based on a priori hypotheses. We observed that, in both datasets, around three quarters of the *APOE-ε4* carriership effect on MTL tau was mediated by amyloid-β (ROS/MAP: PM=79.5%; AZSAND: PM=74.5%) and the direct effect was no longer significant (ROS/MAP: *p*=0.436; AZSAND: *p*=0.124), which suggests a complete mediation. In this case, all other paths were significant in the ROS/MAP dataset and all except one (neocortical neuritic → MTL tau: *p*=0.209) in the AZSAND dataset, supporting the use of the Model 3 in our previous analysis.

On the other hand, less than half of the female sex effect on MTL tau was mediated by amyloid-β measures (ROS/MAP: PM=44.0%; AZSAND: PM=14.4%), and the independent effect (i.e., effect after taking into account amyloid-β-mediated effects) was still significant in both cohorts (ROS/MAP: p=0.002; AZSAND: p=0.001). These mediation effects were similarly weighted in three different paths in the ROS/MAP dataset (neocortical neuritic → MTL tau: 14.4%, MTL neuritic → MTL tau: 12.8%, and neocortical neuritic → MTL neuritic → MTL tau: 13.8%, Supplementary Table 2). In the case of the AZSAND dataset, only the sequential path (neocortical neuritic → MTL neuritic → MTL tau: 28.9%) was significant. The specific weights of all these paths are shown in Supplementary Table 2.

Overall, the *APOE-ε4* carriership effect on MTL tau was mediated by higher levels of amyloid-β pathology in the MTL with or without the effect of neocortex amyloid-β pathology in both cohorts. In contrast, the sex effect on MTL tau was partially independent on amyloid-β pathology levels.

#### Sensitivity analyses

**Fig. 6:**
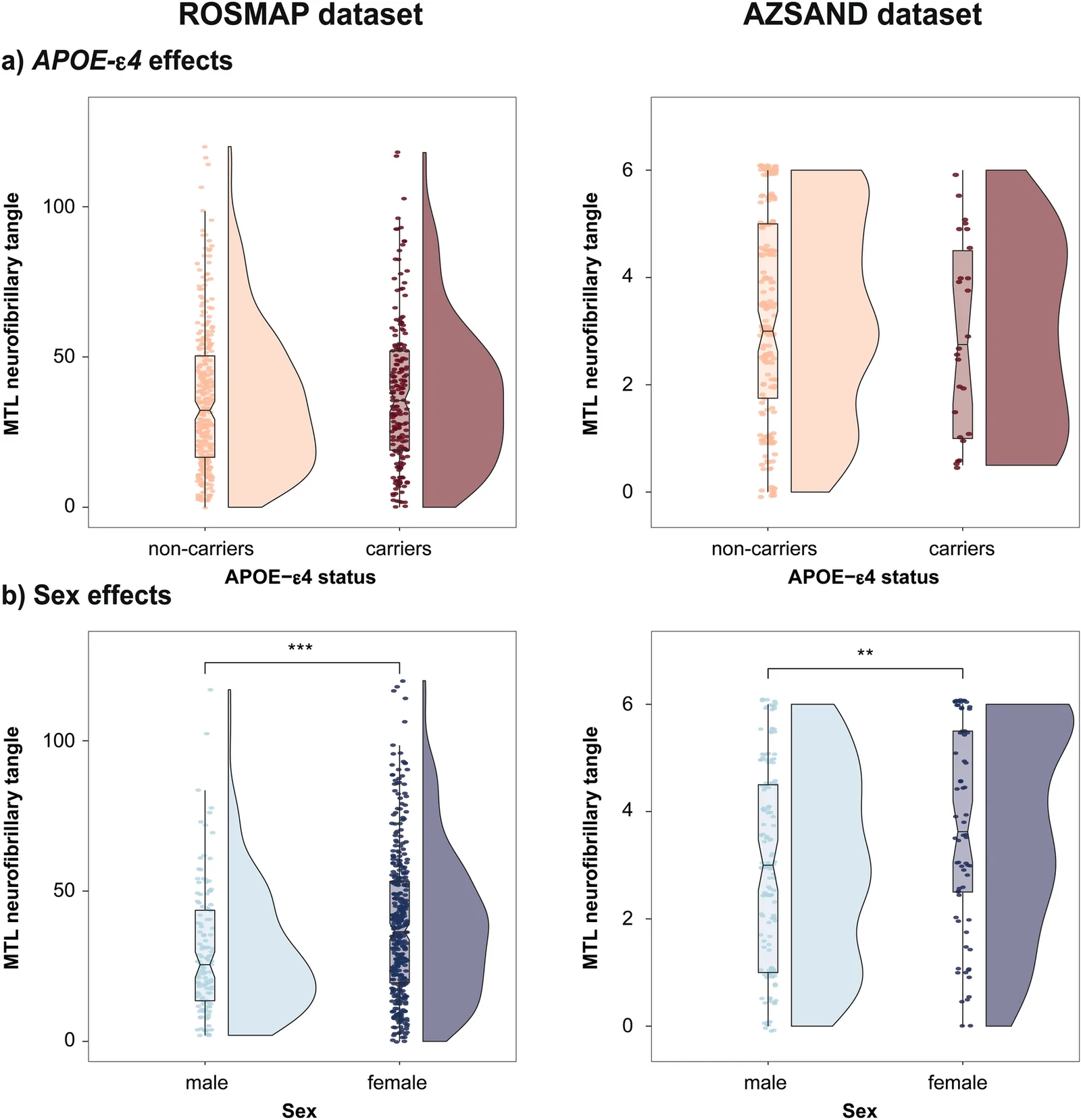
Total effects of *APOE-ε4* carriership and female sex on MTL tau in participants without global amyloid-β pathology. *APOE-ε4* carriership (**A**) and female sex (**B**) effects on MTL neurofibrillary tau tangles in ROS/MAP (first column) and AZSAND (second column) datasets only in participants with none score (lowest) on the CERAD scale. Both effects are calculated from the same model. P-values were calculated adjusting for age and presence of other co-pathologies (ROS/MAP: CAA, LBS and atherosclerosis; AZSAND: CAA and LBS). Differences in scale are due to fully quantitative (ROS/MAP) and semi-quantitative (AZSAND) methods of pathology measurement. ***p<0.001. AZSAND, Arizona Study of Aging and Neurodegenerative Disorders; CAA, cerebral amyloid angiopathy; LB, Lewy-body pathology; MTL, medial temporal lobe; ROS/MAP, Religious Orders Study and Rush Memory and Aging Project

As an additional analysis, we investigated whether the differences observed in MTL tau by *APOE-ε4* carriership and sex were also observed in participants without significant amyloid-β pathology. To this end, we restricted our analyses to participants with score equal to *none* (lowest) on the CERAD scale (ROS/MAP: n=499; AZSAND: n=211; Supplementary Table 3). As expected based on our previous analyses, *APOE-ε4* carriership effects on MTL tau were not significant in this population (ROS/MAP: β[95% CI]=0.02 [− 0.17, 0.21], *p*=0.813; AZSAND: β[95% CI]=− 0.15 [− 0.52, 0.22], *p*=0.421), whereas female sex effects remained significant in ROS/MAP (β[95% CI]=0.35 [0.15, 0.55], *p*<0.001) and showed a trend in AZSAND (β[95% CI]=0.23 [− 0.03, 0.48], *p*=0.077, Fig. 6).

We additionally investigated *APOE-ε4* effects separately in females and males. *APOE-ε4* was associated with greater MTL tau in both sexes before accounting for amyloid-β (all *p*≤0.023), but these associations became non-significant after adjustment for amyloid-β (all *p*≥0.087). Consistently, the *APOE-ε4* × sex interaction was not significant after accounting for amyloid-β in either cohort (ROS/MAP: *p*=0.625; AZSAND: *p*=0.209).

We also repeated the main analyses separately for the entorhinal cortex and hippocampus. Results were consistent with the MTL composite analyses: after accounting for amyloid-β, female sex remained significantly associated with tau in both regions and cohorts (all *p*≤0.026), whereas *APOE-ε4* effects were no longer significant (all *p*≥0.10). Before amyloid-β adjustment, results were also consistent (Supplementary Table 5).

We also repeated the optimal models (i.e., ROS/MAP: adjusted for neocortical diffuse and neuritic amyloid-β plaques, and MTL neuritic plaques; AZSAND: adjusted for MTL amyloid-β) while additionally adjusting for clinical stage or neocortical tau, to account for disease stage. When adjusting for clinical stage, the results for sex remained the same, with female sex having a significant effect even when adjusting for all amyloid-β measures (ROS/MAP: β[95% CI]=0.14 [0.05, 0.23], *p*=0.003; AZSAND: β[95% CI]=0.18 [0.09, 0.28], *p*<0.001). When adjusting for clinical stage, the effect of *APOE-ε4* carriership became non-significant (ROS/MAP: β[95% CI]=0.06 [− 0.04, 0.17], *p*=0.249; AZSAND: β[95% CI]=0.04 [− 0.07, 0.14], *p*=0.518, Supplementary Table 5). Similar results were observed when adjusting for neocortical tau, both for female sex (ROS/MAP: β[95% CI]=0.11 [0.02, 0.20], *p*=0.012; AZSAND: β[95% CI]=0.12 [0.03, 0.21], *p*=0.012) and *APOE-ε4* carriership (ROS/MAP: β[95% CI]=0.05 [− 0.05, 0.16], p=0.310; AZSAND: β[95% CI]=0.02 [− 0.08, 0.13], *p*=0.694, Supplementary Table 6). Notably, the effect of MTL amyloid-β on MTL tau burden remained significant even when adjusting for clinical stages (ROS/MAP: β[95% CI]=0.29 [0.23, 0.35], *p*<0.001; AZSAND: β[95% CI]=0.44 [0.33, 0.55], *p*<0.001) and neocortical tau (ROS/MAP: β[95% CI]=0.27 [0.20, 0.33], p<0.001; AZSAND: β[95% CI]=0.33 [0.22, 0.44], *p*<0.001; Supplementary Table 7). Similar results were observed when CERAD score or Braak stage were additionally included as covariates: the female sex effect remained significant, whereas the *APOE-ε4* effect remained non-significant in both cohorts (Supplementary Table 8).

## Discussion

In two large, independent neuropathology cohorts, we show that both *APOE-ε4* carriership and female sex are associated with greater tau tangle burden in the MTL. In *APOE-ε4* carriers, the elevated MTL tau burden was largely accounted for by higher amyloid-β burden. Adjustment for neocortical amyloid-β substantially attenuated the *APOE-ε4*–tau association, with regional MTL amyloid-β providing additional explanatory information. By contrast, the higher MTL tau burden observed in females was largely independent of both global and MTL amyloid-β pathology. Altogether, our study suggests that these two risk factors contribute to MTL tau through distinct pathways [74].

Our observation that females exhibit higher tau levels is in line with several previous studies in humans [16, 30, 37, 54, 55] and animal models [44, 73]. Importantly, our neuropathology-based findings extend this literature by showing that female-associated elevations in MTL tau are not fully explained by either global or regional amyloid-β burden. This pattern is consistent with models in which sex-related factors modulate early tau vulnerability in the MTL, potentially contributing to limbic-predominant patterns that are reported more frequently in women [29, 52, 69]. Mechanistically, endocrine transitions, particularly menopause-related estrogen loss, have been linked to tau burden [17, 23, 75], and sex differences in neuroimmune biology and microglial activation have been associated with both AD proteinopathies [20, 35]. While our data cannot adjudicate mechanisms, the persistence of the sex effect in low amyloid-β participants and after adjustment for disease stage proxies suggests that female-associated MTL tau accumulation is at least partly independent of amyloid-β. Higher MTL tau burden in females may accelerate disease progression, and thereby increase vulnerability to larger tau accumulation and tau-mediated cognitive decline [18, 24, 64]. Future work to define the biological basis of regional tau vulnerability in women will be essential for personalized treatment strategies.

Our findings also refine the interpretation of *APOE-ε4*–tau associations. Prior work has produced mixed conclusions regarding whether *APOE-ε4* influences tau independently of amyloid-β, with some neuroimaging studies suggesting a preferential *APOE-ε4* association with MTL tau independent of neocortical amyloid-β [59, 68], whereas others support a dominant amyloid-β-mediated pathway [22, 32, 42, 47, 72]. Using neuropathology, the reference standard for quantifying regional plaques and tangles, we found that the higher MTL tau burden in *APOE-ε4* carriers was largely explained by elevated amyloid-β levels, consistent with the view that *APOE-ε4* amplifies tau burden primarily through its strong effects on amyloid-β accumulation and distribution [22, 39, 41, 45]. This interpretation is further supported by our sensitivity analyses showing no significant APOE-ε4 association with MTL tau in the subset with minimal neuritic plaques [28, 30], and by the attenuation of *APOE-ε4* effects when controlling for disease stage proxies. Together, these findings support a predominantly amyloid-β-mediated association between *APOE-ε4* and MTL tau in these neuropathology cohorts.

An advantage of our neuropathological approach is the ability to quantify amyloid-β regionally within the MTL, in addition to neocortical amyloid-β. Many in vivo studies quantify amyloid-β using a global neocortical composite with PET, which may obscure the contribution of regional amyloid-β, particularly within the MTL. Our results highlight that incorporating regional amyloid-β measures can sharpen mechanistic inference about tau vulnerability and may reconcile discrepancies across studies that differ in how amyloid-β is operationalized. Notably, this effect remained significant even after accounting for clinical disease stage or neocortical tau, indicating that MTL amyloid-β contributes to local tau pathology beyond simply reflecting more advanced disease. This point may be especially important in early phases of disease, when localized amyloid-β and tau deposition may be present below the detection thresholds of PET [33, 34, 53], and when off-target binding and limited spatial resolution complicate interpretation of MTL tau-PET signals [34]. Therefore, the use of neuropathological data in this study was essential to reliably capture the regional relationship between amyloid-β and tau pathologies within the MTL.

These findings may help generate testable hypotheses for therapeutic studies and trial methodology. In our autopsy cohorts, the *APOE-ε4* association with MTL tau appeared largely accounted for by amyloid-β measures, which suggests that amyloid-β-related processes may be a major correlate of *APOE-ε4*-linked tau burden in early regions. Whether modifying amyloid-β alters subsequent tau accumulation in a genotype-dependent manner cannot be inferred from the present design and will require longitudinal and interventional evidence. By contrast, the female-associated elevation in MTL tau persisted after accounting for global and regional amyloid-β, indicating that amyloid-β burden, as operationalized here, does not fully explain sex differences in MTL tau pathology. Post hoc subgroup findings from some anti-amyloid-β trials have suggested potential sex differences in outcomes, but these analyses were exploratory and not powered for definitive interaction testing [19, 26]. Collectively, these results support considering sex and *APOE* genotype in prespecified analytic plans for trials that aim to target amyloid-β, tau, or *APOE*-related pathways [25, 46, 62, 71].

A strength of this study is the use of neuropathology, which enabled sensitive, region-specific quantification of plaques and tangles in the MTL, overcoming limitations of PET in terms of resolution and detection limits. Second, we accounted for common co-pathologies, an advantage over in vivo studies that currently lack reliable biomarkers for some of them. Third, we replicated findings in two independent neuropathology cohorts, supporting robustness. However, our study also has some limitations. First, the generalizability of our findings is limited by the lack of racial, ethnic, and socioeconomic diversity of our cohorts. Second, although we adjusted for several co-pathologies, we did not model other sex- and *APOE*-associated processes such as systemic inflammation, cardiovascular health, endocrine and resilience factors, which may influence MTL vulnerability. Third, neuropathological measures may be incomplete, especially when using semi-quantitative measures, such as those used in the AZSAND dataset. Additionally, methodological differences between cohorts may have influenced results. In particular, tau pathology was assessed using different methods across cohorts. AT8 immunohistochemistry, used in ROS/MAP, and Gallyas silver staining, used in AZSAND, detect overlapping but not identical aspects of tau pathology, with AT8 detecting earlier phosphorylated tau pathology and Gallyas preferentially detecting fibrillar/argyrophilic pathology [13]. Nonetheless, the observation of similar associations in both cohorts despite the use of different histopathological methods supports the robustness of our main findings. Also, the cross-sectional nature of autopsy data precludes causal inference about temporal ordering. Longitudinal studies tracking regional amyloid-β and tau accumulation over time will be necessary to confirm these mechanisms. Lastly, we assessed biological sex, which does not account for the potential influences of gender-related psychosocial and cultural factors on AD vulnerability. Future studies should explore the distinct contributions of biological sex vs. perceived gender to tau accumulation, an area that remains largely unexplored in AD research.

## Conclusion

Both *APOE-ε4* carriers and females exhibited greater tau burden in the MTL across two independent neuropathology cohorts. In carriers, the association with MTL tau was largely explained by amyloid-β measures, including regional MTL amyloid-β, whereas the female-associated elevation in MTL tau persisted after accounting for global and regional amyloid-β. Together, these findings are consistent with at least partially distinct correlates of MTL tau burden for *APOE-ε4* and sex. Future longitudinal in vivo studies will be important to determine temporal relationships among regional amyloid-β and tau pathologies and to evaluate whether these associations translate into differential therapeutic responsiveness. Further research into why females are more vulnerable to MTL tau deposition may clarify sex differences in AD prevalence and progression and guide precision-medicine trial designs.

## Role of the funder/sponsor

The funding sources had no role in the design and conduct of the study; in the collection, analysis, interpretation of the data; or in the preparation, review, or approval of the manuscript.

## Competing interests

GS has received speaker fees from Springer, GE Healthcare, Biogen, Esteve, Adium and Alamar and she serves on the advisory board of Johnson&Johnson. R.O. is currently a full-time employee of Eli Lilly and Company. His contribution to the work presented in this manuscript was performed as an employee of Amsterdam University Medical Centers and Lund University. R.O. has received research funding/support from European Research Council, ZonMw, NWO, National Institute of Health, Alzheimer Association, Alzheimer Nederland, Stichting Dioraphte, Cure Alzheimer’s fund, Health Holland, ERA PerMed, Alzheimerfonden, Hjarnfonden, Avid Radiopharmaceuticals, Janssen Research & Development, Roche, Quanterix and Optina Diagnostics, has given lectures in symposia sponsored by GE Healthcare, received speaker fees from Springer, and was an advisory board/steering committee member for Asceneuron, Biogen, Johnson & Johnson and Bristol Myers Squibb. All the aforementioned has been paid to Amsterdam University Medical Centers and Lund University. OH is employed by Eli Lilly and Lund University.

## Supplementary Information

Below is the link to the electronic supplementary material.

Supplementary Material 1.

## Data Availability

Anonymized data will be shared by request from a qualified academic investigator for the sole purpose of replicating procedures and results presented in the article.
