# Supplementary material for: Global and regional amyloid-β in explaining APOE-ε4 and sex associations with medial temporal tau: a neuropathology study
Source: Acta Neuropathol. 2026 Oct 9;152(1):49. doi: 10.1007/s00401-026-03095-2 (PMC13652906; doi:10.1007/s00401-026-03095-2)
Supplement: Supplementary file 1 [file 401_2026_3095_MOESM1_ESM.docx]

**Global and regional amyloid-β in explaining APOE-ε4 and sex associations with medial temporal tau: A neuropathology study**

Gemma Salvadó, Colin Groot, Laura E. Wisse, Jacob W. Vogel, Thomas G. Beach, Geidy E. Serrano, Eric M. Reiman, Julie A. Schneider, David A. Bennett, Rik Ossenkoppele, Oskar Hansson

**Table of contents**

**Tables**

**Supplementary table 1: Total effects of sex and *APOE-ε4* carriership on amyloid-β measures**

**Supplementary table 2: Direct and indirect effects of sex and *APOE-ε4* carriership on tau in the medial temporal lobe**

**Supplementary Table 3: Demographic characteristics by cohort in participants with low amyloid-β pathology**

**Supplementary Table 4:** ***APOE-ε4* carriership and sex effects on MTL tau for participants with low levels of amyloid-β pathology**

**Supplementary Table 5:** ***APOE-ε4* carriership and sex effects on ERC and hippocampal regions separately**

**Supplementary table 6: Total effects of sex and *APOE-ε4* carriership after adjustment by clinical stage or neocortical tau**

**Supplementary table 7: Total effects of neocortical and MTL amyloid-β measures on MTL tau after adjustment by clinical stage or neocortical tau**

**Supplementary table 8: Total effects of sex and *APOE-ε4* carriership on MTL tau after adjustment by CERAD or Braak stages**

|  | ROS/MAP dataset | | | | | AZSAND dataset | | | | |
| --- | --- | --- | --- | --- | --- | --- | --- | --- | --- | --- |
|  | Sex | | *APOE-ε4* carriership | | R^2^ | Sex | | *APOE-ε4* carriership | | R^2^ |
|  | β [95%CI] | p | β [95%CI] | p |  | β [95%CI] | p | β [95%CI] | p |  |
| Neuritic neocortical amyloid-β plaques | 0.25  [0.15, 0.35] | <0.001 | 0.55  [0.44, 0.67] | <0.001 | 0.14 | 0.12  [0.02, 0.22] | 0.017 | 0.48  [0.37, 0.59] | <0.001 | 0.40 |
| Neuritic MTL amyloid-β plaques | 0.32  [0.22, 0.42] | <0.001 | 0.59  [0.48, 0.70] | <0.001 | 0.13 | 0.07  [-0.03, 0.171] | 0.178 | 0.51  [0.40, 0.62] | <0.001 | 0.38 |
| Neuritic MTL amyloid-β plaques (adjusted for neuritic neocortical amyloid-β plaques) | 0.15  [0.07, 0.22] | <0.001 | 0.20  [0.12, 0.28] | <0.001 | 0.56 | -0.03  [-0.09, 0.02] | 0.214 | 0.10  [0.04, 0.16] | 0.002 | 0.82 |

**Supplementary table 1: Total effects of sex and *APOE-ε4* carriership on amyloid-β measures**

All models include age and presence of co-pathologies as covariates (ROS/MAP: CAA, LB and atherosclerosis and, AZSAND: CAA and LB). Each row is a unique model including both sex and *APOE-ε4* carriership as predictors in the same model, per cohort. Men and *APOE-ε4* non-carriers are the reference group. R^2^ is adjusted for the number of terms in the model.

Abbreviations: AZSAND, Arizona Study of Aging and Neurodegenerative Disorders; CAA, cerebral amyloid angiopathy; CI, confidence interval; LB, Lewy-body pathology; MTL, medial temporal lobe; ROS/MAP, Religious Orders Study and Rush Memory and Aging Project.

|  | ROS/MAP dataset | | | | | | AZSAND dataset | | | | | |
| --- | --- | --- | --- | --- | --- | --- | --- | --- | --- | --- | --- | --- |
|  | Sex | | | *APOE-ε4* carriership | | | Sex | | | *APOE-ε4* carriership | | |
|  | β [95%CI] | PM | p | β [95%CI] | PM | p | β [95%CI] | PM | p | β [95%CI] | PM | p |
| Total | 0.125  [0.082, 0.169] | - | <0.001 | 0.149  [0.096, 0.201] | - | <0.001 | 0.097  [0.044, 0.150] | - | <0.001 | 0.153  [0.097, 0.210] | - | <0.001 |
| Direct | 0.067  [0.025, 0.109] | 53.6 | 0.002 | 0.020  [-0.030, 0.069] | 13.4 | 0.436 | 0.083  [0.036, 0.131] | 85.6 | 0.001 | 0.039  [-0.011, 0.089] | 25.5 | 0.124 |
| NC-dAβ | 0.003  [-0.004, 0.010] | 2.4 | 0.432 | 0.033  [0.024, 0.042] | 22.1 | <0.001 | - | - | - | - | - | - |
| NC-nAβ | 0.018  [0.007, 0.029] | 14.4 | 0.001 | 0.027  [0.012, 0.043] | 18.1 | <0.001 | -0.005  [-0.014, 0.004] | -5.2 | 0.288 | -0.020  [-0.051, 0.011] | -13.1 | 0.209 |
| MTL-nAβ | 0.016  [0.008, 0.024] | 12.8 | <0.001 | 0.012  [0.002, 0.022] | 8.1 | 0.019 | -0.009  [-0.025, 0.006] | -9.3 | 0.234 | 0.026  [0.008, 0.045] | 17.0 | 0.006 |
| NC-dAβ→NC-nAβ | 0.000  [-0.001, 0.002] | 0.0 | 0.445 | 0.005  [0.003, 0.008] | 3.4 | <0.001 | - | - | - | - | - | - |
| NC-dAβ→MTL-nAβ | 0.001  [-0.001, 0.003] | 0.8 | 0.433 | 0.012  [0.007, 0.017] | 8.1 | <0.001 | - | - | - | - | - | - |
| NC-dAβ→NC-nAβ→  MTL-nAβ | 0.000  [-0.001, 0.001] | 0.0 | 0.433 | 0.005  [0.003, 0.007] | 3.4 | <0.001 | - | - | - | - | - | - |
| NC-nAβ→MTL-nAβ | 0.017  [0.009, 0.025] | 13.6 | <0.001 | 0.026  [0.015, 0.036] | 17.4 | <0.001 | 0.028  [0.005, 0.051] | 28.9 | 0.020 | 0.108  [0.073, 0.142] | 70.6 | <0.001 |

**Supplementary table 2: Direct and indirect effects of sex and *APOE-ε4* carriership on tau in the medial temporal lobe**

For each dataset, a unique model was constructed including both sex and *APOE-ε4* carriership as predictors, all amyloid-β measures as endogenous variables, and age and presence of co-pathologies as covariates (ROS/MAP: CAA, LB and atherosclerosis and, AZSAND: CAA and LB). Men and *APOE-ε4* non-carriers are the reference group. Percentage mediation (PM) effect was calculated as the percentage of the total effect that was mediated by each amyloid-β measure. All possible paths in each model are shown in the table.

Abbreviations: AZSAND, Arizona Study of Aging and Neurodegenerative Disorders; CAA, cerebral amyloid angiopathy; CI, confidence interval; dAβ, diffuse amyloid-β plaques; LB, Lewy-body pathology; MTL, medial temporal lobe; nAβ, neuritic amyloid-β plaques; NC, neocortical; ROS/MAP, Religious Orders Study and Rush Memory and Aging Project.

|  | ROS/MAP dataset | | | | | | AZSAND dataset | | | | |
| --- | --- | --- | --- | --- | --- | --- | --- | --- | --- | --- | --- |
|  | **Overall (N=499)** | **Male (N=511)** | **Female (N=1,076)** | ***APOE-ε4* Non-carriers (N=310)** | ***APOE-ε4* Carriers (N=189)** | **Overall (N=211)** | | **Male (N=141)** | **Female (N=70)** | ***APOE-ε4* Non-carriers (N=186)** | ***APOE-ε4* Carriers (N=25)** |
| Age, mean (SD) | 90.3 (5.9) | 89.2 (5.3) | 90.6 (6.1) | 91.4 (5.6) | 88.5 (6.0) | 79.3 (10.5) | | 78.2 (10.4) | 81.7 (10.3) | 79.4 (10.7) | 78.8 (9.19) |
| Female, n (%) | 375 (75.2%) | - | 1076 (100%) | 236 (76.1%) | 139 (73.5%) | 70 (33.2%) | | - | 70 (100%) | 62 (33.3%) | 8 (32.0%) |
| *APOE-ε4* carriership, n (%) | 189 (37.9%) | 50  (40.3%) | 139 (37.1%) | - | 25  (100%) | 25 (11.8%) | | 17 (12.1%) | 8 (11.4%) | - | 25  (100%) |
| Diagnosis, n (%) | | | | | | | | | | | |
| CU | 83 (16.6%) | 19 (15.3%) | 64 (17.1%) | 65 (21.0%) | 18 (9.5%) | 80 (37.9%) | | 47 (33.3%) | 33 (47.1%) | 71 (38.2%) | 9 (36.0%) |
| MCI | 92 (18.4%) | 27 (21.8%) | 65 (17.3%) | 65 (21.0%) | 27 (14.3%) | 15 (7.1%) | | 11 (7.8%) | 4 (5.7%) | 13 (7.0%) | 2 (8.0%) |
| AD | 320 (64.1%) | 77  (62.1%) | 243 (64.8%) | 177 (57.1%) | 143 (75.7%) | 1 (0.5%) | | 1 (0.7%) | 0 (0%) | 1 (0.5%) | 0 (0%) |
| Other | 4 (0.8%) | 1 (0.8%) | 3 (0.8%) | 3 (1.0%) | 1 (0.5%) | 115 (54.5%) | | 82 (58.1%) | 33 (47.1%) | 101  (54.3%) | 14  (56.0%) |
| Neuropathologic measures | | | | | | | | | | | |
| Neocortical diffuse amyloid-β plaques, mean (SD)† | 19.5 (12.5) | 18.6 (10.8) | 19.7 (13.1) | 18.2 (11.7) | 21.5 (13.7) | - | | - | - | - | - |
| Neocortical amyloid-β plaques, mean (SD) | 23.8 (9.6) | 22.5 (9.0) | 24.2 (9.7) | 22.6 (8.10) | 25.7 (11.4) | 0.193 (0.844) | | 0.19 (0.93) | 0.20 (0.64) | 0.16 (0.80) | 0.42 (1.14) |
| MTL neuritic amyloid-β plaques, mean (SD) | 15.3 (8.8) | 12.7 (7.7) | 16.2 (9.0) | 14.1 (7.82) | 17.3 (9.94) | 0.08 (0.47) | | 0.10 (0.54) | 0.04 (0.27) | 0.05 (0.34) | 0.30 (0.97) |
| MTL neurofibrillary tangles, mean (SD) | 36.6 (24.1) | 30.0 (21.6) | 38.7 (24.5) | 35.9 (23.9) | 37.7 (24.4) | 3.25 (1.88) | | 3.01 (1.82) | 3.73 (1.92) | 3.3 (1.9) | 2.9 (1.8) |
| CAA, n (%) | 245 (49.1%) | 64  (51.6%) | 181 (48.3%) | 121 (39.0%) | 124 (65.6%) | 20 (9.5%) | | 15 (10.6%) | 5 (7.1%) | 17 (9.1%) | 3 (12.0%) |
| LB, n (%) | 125 (25.1%) | 30 (24.2%) | 95 (25.3%) | 75 (24.2%) | 50 (26.5%) | 25 (11.8%) | | 15 (10.6%) | 10 (14.3%) | 20 (10.8%) | 5 (20.0%) |
| Arteriosclerosis, n (%) | 143 (28.7%) | 28  (22.6%) | 115 (30.7%) | 88  (28.4%) | 55  (29.1%) | - | | - | - | - | - |
| TDP-43, n(%)* | 224 (47.0%) | 46 (40.7%) | 178 (48.9%) | 125 (41.7%) | 99 (55.9%) | 16 (14.3%) | | 10 (14.1%) | 6 (14.6%) | 14 (13.7%) | 2 (20.0%) |

**Supplementary Table 3: Demographic characteristics by cohort in participants with low amyloid-β pathology**

† Ten cases missing in ROS/MAP

* 22 cases missing in ROS/MAP and 99 cases missing in AZSAND

Low amyloid-β pathology was defined as CERAD score as none in both ROS/MAP and AZSAND.

Abbreviations: AD, Alzheimer’s dementia; AZSAND, Arizona Study of Aging and Neurodegenerative Disorders; CAA, cerebral amyloid angiopathy; CU, cognitively unimpaired; LBS, Lewy-body pathology; MCI, mild cognitive impairment; MTL, medial temporal lobe; ROS/MAP, Religious Orders Study and Rush Memory and Aging Project.

| Dataset | Female sex | | *APOE-ε4* carriership | | R^2^ | AIC |
| --- | --- | --- | --- | --- | --- | --- |
|  | β [95%CI] | p | β [95%CI] | p |  |  |
| ROS/MAP | 0.35 [0.15, 0.55] | <0.001 | 0.02 [-0.17, 0.21] | 0.813 | 0.04 | 1403 |
| AZSAND | 0.23 [-0.03, 0.48] | 0.077 | -0.15 [-0.52, 0.22] | 0.421 | 0.25 | 547 |

**Supplementary Table 4:** ***APOE-ε4* carriership and sex effects on MTL tau for participants with low levels of amyloid-β pathology**

This table shows the total sex and *APOE-ε4* carriership effect (only adjusted for covariates) on MTL tau in participants with low levels of amyloid-β pathology. Low amyloid-β pathology was defined as CERAD score as none in both ROS/MAP and AZSAND. All models include age and presence of co-pathologies as covariates (ROS/MAP: CAA, LBS and atherosclerosis and, AZSAND: CAA and LBS). Men and *APOE-ε4* non-carriers are the reference group. R^2^ is adjusted for the number of terms in the model.

Abbreviations: AIC, Akaike criteria; AZSAND, Arizona Study of Aging and Neurodegenerative Disorders; CAA, cerebral amyloid angiopathy; CI, confidence interval; LBS, Lewy-body syndrome; MTL, medial temporal lobe; nft, neurofibrillary tangles; ROS/MAP, Religious Orders Study and Rush Memory and Aging Project.

|  | ROS/MAP dataset | | | | | | | |
| --- | --- | --- | --- | --- | --- | --- | --- | --- |
|  | Basic model | | | | Full model | | | |
|  | Sex | | *APOE-ε4* carriership | | Sex | | *APOE-ε4* carriership | |
| Region of interest | β [95%CI] | p | β [95%CI] | p | β [95%CI] | p | β [95%CI] | p |
| ERC | 0.24 [0.13, 0.34] | <0.001 | 0.34 [0.24, 0.47] | <0.001 | 0.11 [0.01, 0.20] | 0.026 | 0.06 [-0.05, 0.17] | 0.273 |
| Hippocampus | 0.23 [0.13, 0.34] | <0.001 | 0.33 [0.22, 0.45] | <0.001 | 0.12 [0.03, 0.22] | 0.013 | 0.09 [-0.02, 0.22] | 0.104 |
|  | AZSAND dataset | | | | | | | |
| ERC | 0.16 [0.05, 0.27] | 0.005 | 0.29 [0.17, 0.41] | <0.001 | 0.13 [0.02, 0.23] | 0.015 | 0.07 [-0.04, 0.19] | 0.228 |
| Hippocampus | 0.21 [0.10, 0.32] | <0.001 | 0.31 [0.19, 0.43] | <0.001 | 0.19 [0.09, 0.29] | <0.001 | 0.08 [-0.03, 0.20] | 0.165 |

**Supplementary table 5: *APOE-ε4* carriership and sex effects on ERC and hippocampal regions separately**

All models include age and presence of co-pathologies as covariates (ROS/MAP: CAA, LB and atherosclerosis and, AZSAND: CAA and LB). Full model additionally includes neuritic neocortical amyloid-β plaques and neuritic MTL amyloid-β plaques, as covariates. And in ROS/MAP cohort full model also included diffuse amyloid-β plaques as additional covariate. Each row is a unique model including both sex and *APOE-ε4* carriership as predictors in the same model, per cohort. Men and *APOE-ε4* non-carriers are the reference group.

Abbreviations: AZSAND, Arizona Study of Aging and Neurodegenerative Disorders; CAA, cerebral amyloid angiopathy; CI, confidence interval; LB, Lewy-body pathology; MTL, medial temporal lobe; ROS/MAP, Religious Orders Study and Rush Memory and Aging Project.

|  | ROS/MAP dataset | | | | | AZSAND dataset | | | | |
| --- | --- | --- | --- | --- | --- | --- | --- | --- | --- | --- |
|  | Sex | | *APOE-ε4* carriership | | R^2^ | Sex | | *APOE-ε4* carriership | | R^2^ |
| Model | β [95%CI] | p | β [95%CI] | p |  | β [95%CI] | p | β [95%CI] | p |  |
| Adjusted for clinical stages | | | | | | | | | | |
| Neuritic neocortical amyloid-β plaques | 0.24  [0.15, 0.33] | <0.001 | 0.42  [0.31, 0.53] | <0.001 | 0.22 | 0.10  [0.01, 0.19] | 0.033 | 0.26  [0.16, 0.36] | <0.001 | 0.54 |
| Neuritic MTL amyloid-β plaques | 0.31  [0.21, 0.41] | <0.001 | 0.48  [0.37, 0.60] | <0.001 | 0.19 | 0.03  [-0.05, 0.12] | 0.448 | 0.28  [0.18, 0.38] | <0.001 | 0.54 |
| Neuritic MTL amyloid-β plaques (adjusted for neuritic neocortical amyloid-β plaques) | 0.14  [0.07, 0.21] | <0.001 | 0.19  [0.10, 0.27] | <0.001 | 0.56 | -0.04  [-0.09, 0.01] | 0.126 | 0.07  [0.01, 0.13] | 0.025 | 0.83 |
| MTL tau | 0.26  [0.17, 0.36] | <0.001 | 0.26  [0.15, 0.38] | <0.001 | 0.17 | 0.19  [0.09, 0.29] | <0.001 | 0.13  [0.02, 0.24] | 0.026 | 0.42 |
| MTL tau (adjusted for neuritic neocortical amyloid-β plaques) | 0.18  [0.09, 0.27] | <0.001 | 0.12  [0.01, 0.23] | 0.035 | 0.26 | 0.16  [0.07, 0.26] | 0.001 | 0.07  [-0.04, 0.18] | 0.241 | 0.45 |
| MTL tau (adjusted for neuritic MTL amyloid-β plaques) | 0.14  [0.05, 0.24] | 0.003 | 0.08  [-0.03, 0.18] | 0.159 | 0.29 | 0.17  [0.08, 0.27] | 0.001 | 0.03  [-0.08, 0.14] | 0.575 | 0.48 |
| MTL tau (adjusted for neuritic neocortical and MTL amyloid-β plaques)* | 0.14  [0.05, 0.23] | 0.003 | 0.06  [-0.04, 0.17] | 0.249 | 0.29 | 0.18  [0.09, 0.28] | <0.001 | 0.04  [-0.07, 0.14] | 0.518 | 0.48 |
| Adjusted for neocortical tau | | | | | | | | | | |
| Neuritic neocortical amyloid-β plaques | 0.14  [0.06, 0.23] | <0.001 | 0.34  [0.23, 0.44] | <0.001 | 0.38 | 0.06  [-0.03, 0.15] | 0.226 | 0.30  [0.20, 0.40] | <0.001 | 0.52 |
| Neuritic MTL amyloid-β plaques | 0.23  [0.14, 0.32] | <0.001 | 0.41  [0.31, 0.52] | <0.001 | 0.29 | -0.01  [-0.10, 0.08] | 0.796 | 0.29  [0.20, 0.39] | <0.001 | 0.55 |
| Neuritic MTL amyloid-β plaques (adjusted for neuritic neocortical amyloid-β plaques) | 0.14  [0.06, 0.21] | <0.001 | 0.19  [0.10, 0.27] | <0.001 | 0.57 | -0.05  [-0.11, -0.00] | 0.043 | 0.06  [0.00, 0.12] | 0.038 | 0.84 |
| MTL tau | 0.18  [0.09, 0.27] | <0.001 | 0.17  [0.07, 0.27] | 0.001 | 0.29 | 0.11  [0.01, 0.20] | 0.024 | 0.09  [-0.01, 0.20] | 0.091 | 0.48 |
| MTL tau (adjusted for neuritic neocortical amyloid-β plaques) | 0.15  [0.06, 0.24] | 0.001 | 0.10  [-0.0, 0.21] | 0.054 | 0.32 | 0.10  [0.01, 0.19] | 0.037 | 0.04  [-0.06, 0.15] | 0.440 | 0.49 |
| MTL tau (adjusted for neuritic MTL amyloid-β plaques) | 0.11  [0.02, 0.20] | 0.012 | 0.05  [-0.05, 0.16] | 0.291 | 0.35 | 0.11  [0.02, 0.20] | 0.017 | 0.02  [-0.09, 0.12] | 0.763 | 0.51 |
| MTL tau (adjusted for neuritic neocortical and MTL amyloid-β plaques)* | 0.11  [0.02, 0.20] | 0.012 | 0.05  [-0.05, 0.16] | 0.310 | 0.35 | 0.12  [0.03, 0.21] | 0.012 | 0.02  [-0.08, 0.13] | 0.694 | 0.51 |

**Supplementary table 6: Total effects of sex and *APOE-ε4* carriership after adjustment by clinical stage or neocortical tau**

All models include age and presence of co-pathologies as covariates (ROS/MAP: CAA, LB and atherosclerosis and, AZSAND: CAA and LB). Each row is a unique model including both sex and *APOE-ε4* carriership as predictors in the same model, per cohort. Men and *APOE-ε4* non-carriers are the reference group. R^2^ is adjusted for the number of terms in the model. * ROS/MAP cohort also included diffuse amyloid-β plaques as additional covariate.

Abbreviations: AZSAND, Arizona Study of Aging and Neurodegenerative Disorders; CAA, cerebral amyloid angiopathy; CI, confidence interval; LB, Lewy-body pathology; MTL, medial temporal lobe; ROS/MAP, Religious Orders Study and Rush Memory and Aging Project.

|  | ROS/MAP dataset | | | | | AZSAND dataset | | | | |
| --- | --- | --- | --- | --- | --- | --- | --- | --- | --- | --- |
|  | Neocortical amyloid-β | | MTL amyloid-β plaques | | R^2^ | Neocortical amyloid-β | | MTL amyloid-β plaques | | R^2^ |
| Model | β [95%CI] | p | β [95%CI] | p |  | β [95%CI] | p | β [95%CI] | p |  |
| Adjusted for clinical stages | | | | | | | | | | |
| MTL tau (adjusted for neuritic neocortical amyloid-β plaques) | 0.34  [0.30, 0.39] | <0.001 | - | - | 0.26 | 0.23  [0.16, 0.30] | <0.001 | - | - | 0.45 |
| MTL tau (adjusted for neuritic MTL amyloid-β plaques) | - | - | 0.38  [0.34, 0.43] | <0.001 | 0.29 | - | - | 0.35  [0.28, 0.41] | <0.001 | 0.48 |
| MTL tau (adjusted for neuritic neocortical and MTL amyloid-β plaques)* | 0.14  [0.08, 0.21] | <0.001 | 0.29  [0.23, 0.35] | <0.001 | 0.29 | -0.11  [-0.22, -0.00] | 0.043 | 0.44  [0.33, 0.55] | <0.001 | 0.48 |
| Adjusted for neocortical tau | | | | | | | | | | |
| MTL tau (adjusted for neuritic neocortical amyloid-β plaques) | 0.20  [0.15, 0.25] | <0.001 | - | - |  | 0.16  [0.10, 0.23] | <0.001 | - | - | 0.49 |
| MTL tau (adjusted for neuritic MTL amyloid-β plaques) | - | - | 0.28  [0.23, 0.32] | <0.001 | 0.35 | - | - | 0.25  [0.19, 9.32] | <0.001 | 0.51 |
| MTL tau (adjusted for neuritic neocortical and MTL amyloid-β plaques)* | 0.02  [-0.04, 0.09] | 0.529 | 0.27  [0.20, 0.33] | <0.001 | 0.35 | -0.09  [-0.19, 0.02] | 0.106 | 0.33  [0.22, 0.44] | <0.001 | 0.51 |

**Supplementary table 7: Total effects of neocortical and MTL amyloid-β measures on MTL tau after adjustment by clinical stage or neocortical tau**

All models include age and presence of co-pathologies as covariates (ROS/MAP: CAA, LB and atherosclerosis and, AZSAND: CAA and LB). Each row is a unique model including both sex and *APOE-ε4* carriership as predictors in the same model, per cohort. Men and *APOE-ε4* non-carriers are the reference group. R^2^ is adjusted for the number of terms in the model. * ROS/MAP cohort also included diffuse amyloid-β plaques as additional covariate.

Abbreviations: AZSAND, Arizona Study of Aging and Neurodegenerative Disorders; CAA, cerebral amyloid angiopathy; CI, confidence interval; LB, Lewy-body pathology; MTL, medial temporal lobe; ROS/MAP, Religious Orders Study and Rush Memory and Aging Project.

|  | ROS/MAP dataset | | | |
| --- | --- | --- | --- | --- |
|  | Sex | | *APOE-ε4* carriership | |
| Additional covariate | β [95%CI] | p | β [95%CI] | p |
| CERAD | 0.13  [0.04, 0.22] | 0.004 | 0.06  [-0.05, 0.17] | 0.267 |
| Braak stages | 0.10  [0.03, 0.18] | 0.008 | 0.01  [-0.08, 0.09] | 0.863 |
|  | **AZSAND dataset** | | | |
| CERAD | 0.17  [0.07, 0.26] | <0.001 | 0.10  [-0.01, 0.21] | 0.069 |
| Braak stages | 0.08  [0.03, 0.14] | 0.004 | 0.02  [-0.04, 0.09] | 0.474 |

**Supplementary table 8: Total effects of sex and *APOE-ε4* carriership on MTL tau after adjustment by CERAD or Braak stages**

All models include age, neuritic neocortical amyloid-β plaques, neuritic MTL amyloid-β plaques and presence of co-pathologies as covariates (ROS/MAP: CAA, LB and atherosclerosis and, AZSAND: CAA and LB). ROS/MAP cohort also included diffuse amyloid-β plaques as additional covariate. Each row is a unique model including both sex and *APOE-ε4* carriership as predictors in the same model, per cohort. Men and *APOE-ε4* non-carriers are the reference group.

Abbreviations: AZSAND, Arizona Study of Aging and Neurodegenerative Disorders; CAA, cerebral amyloid angiopathy; CI, confidence interval; LB, Lewy-body pathology; MTL, medial temporal lobe; ROS/MAP, Religious Orders Study and Rush Memory and Aging Project.
